# IoMT-enabled implant sensors for early detection of neurovascular complications in craniofacial distraction osteogenesis

**DOI:** 10.1097/MS9.0000000000005118

**Published:** 2026-05-14

**Authors:** Taimoor Ali, Sarum Ali Khan, Zakwan Shahbaz, Rameen Haider, Aiman Tahir, Muhammad Adnan Qamar

**Affiliations:** a Department of Medicine and Surgery, Wah Medical College, Wah Cantt, Pakistan; b Department of Medicine and Surgery, Azra Naheed Medical College, Pakistan; c Department of Medicine and Surgery, Azad Jammu and Kashmir Medical College, Muzaffarabad, AJK, Pakistan; d Department of Internal Medicine, Spinghar Medical University, Kabul, Afghanistan

**Keywords:** biomechanical monitoring, closed-loop feedback, craniofacial distraction osteogenesis, facial nerve injury, implantable sensors, Internet of Medical Things, neurovascular complications

## Abstract

Craniofacial distraction osteogenesis (DO) is an established surgical technique for correcting complex craniofacial deformities and managing upper airway obstruction. Neurovascular complications, including injury to the inferior alveolar, infraorbital, and facial nerves, are recognized risks of the procedure. Current postoperative surveillance depends on periodic clinical examination and interval imaging, neither of which provides continuous insight into the mechanical forces transmitted to adjacent neurovascular structures during the active distraction phase. This letter examines whether implantable sensor technologies integrated with Internet of Medical Things (IoMT) infrastructure could address this monitoring gap. Micro-sensors capable of monitoring strain, pressure, and temperature at the tissue–implant interface have been demonstrated in musculoskeletal applications and are technically transferable to craniofacial distraction hardware. Continuous strain monitoring could detect abnormal force accumulation along the distraction vector before neurovascular injury thresholds are reached. Closed-loop feedback architectures could further allow dynamic modulation of distraction parameters in response to real-time physiological signals, reducing reliance on fixed-schedule activations that do not account for individual tissue response. No clinical studies have yet evaluated IoMT sensor integration in craniofacial DO, and significant barriers remain, including sensor biocompatibility, device miniaturization for pediatric use, wireless signal transmission through bone, and data security. Prospective feasibility studies are required before this approach can be considered for clinical implementation.

*Dear Editor*,

We read with great interest the study by Dong *et al*, which developed and validated a nomogram for predicting in-stent stenosis following pipeline embolization device treatment in unruptured intracranial aneurysms[1]. A central message of that work is that continuous risk stratification and the early detection of implant-related neurovascular complications are essential, as delayed recognition significantly worsens outcomes. This principle, while established in endovascular interventions, is equally relevant in craniofacial distraction osteogenesis (DO), where neurovascular complications are well recognized and where progressive mechanical forces generated by the distraction device act on tissues in close proximity to named neurovascular structures throughout a treatment period typically spanning several weeks.

DO is an established technique for correcting complex craniofacial deformities and managing upper airway obstruction in syndromic craniosynostosis. Nerve injury is a recognized complication, with the inferior alveolar nerve, infraorbital nerve, and branches of the facial nerve all at anatomical risk depending on the distraction site and vector[2]. Facial nerve paresis following mandibular DO has been documented and, while most cases resolve with conservative management or device removal, the onset is often insidious and the clinical presentation variable, making early identification difficult[3].

Implantable sensor technologies integrated with IoMT infrastructure offer a mechanistically grounded approach to closing this gap. Micro-sensors capable of monitoring strain, pressure, and temperature at the tissue–implant interface have been demonstrated in musculoskeletal applications and are technically transferable to craniofacial distraction hardware[4]. Applied in this context, continuous strain monitoring could detect abnormal force accumulation along the distraction vector before it reaches a threshold capable of injuring adjacent nerves or vessels – something periodic clinical review cannot achieve. Furthermore, biosensor systems integrated with closed-loop feedback architectures can dynamically modulate therapeutic parameters in response to real-time physiological signals, raising the possibility of distraction devices that automatically adjust the rate or rhythm of distraction when biomechanical thresholds are approached, reducing dependence on fixed-schedule activations that take no account of individual tissue response[5].

Translating these capabilities into craniofacial DO practice is not without barriers. Long-term biocompatibility of implanted sensors in the craniofacial skeleton, device miniaturization for pediatric applications, reliable wireless signal transmission through bone and soft tissue, and data security within IoMT infrastructure all represent unresolved engineering and regulatory challenges[6].

Critically, no clinical studies have yet evaluated IoMT sensor integration specifically in craniofacial DO, and the proposal advanced here remains a research direction rather than a clinical recommendation. Prospective feasibility studies are needed to determine whether continuous biomechanical monitoring translates into measurable reductions in neurovascular complication rates in this population.

The findings of Dong *et al* reinforce that implant-related neurovascular risk does not end at the operating table. In craniofacial DO, where distraction-phase nerve injury can be insidious and current monitoring tools are reactive rather than continuous, IoMT-enabled implant sensors represent a scientifically coherent avenue for earlier complication detection. Realizing this potential will require dedicated interdisciplinary research and clinical validation before any implementation can be responsibly considered.

## Data Availability

All data used are accessible.
